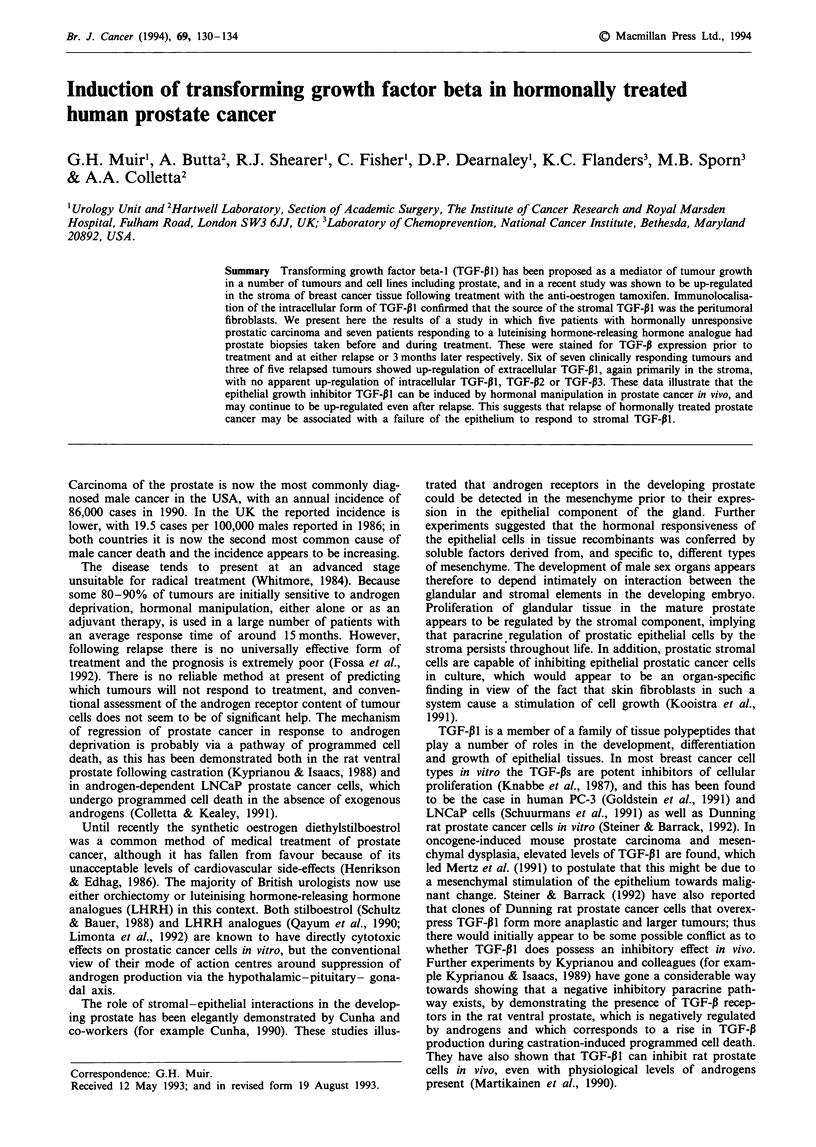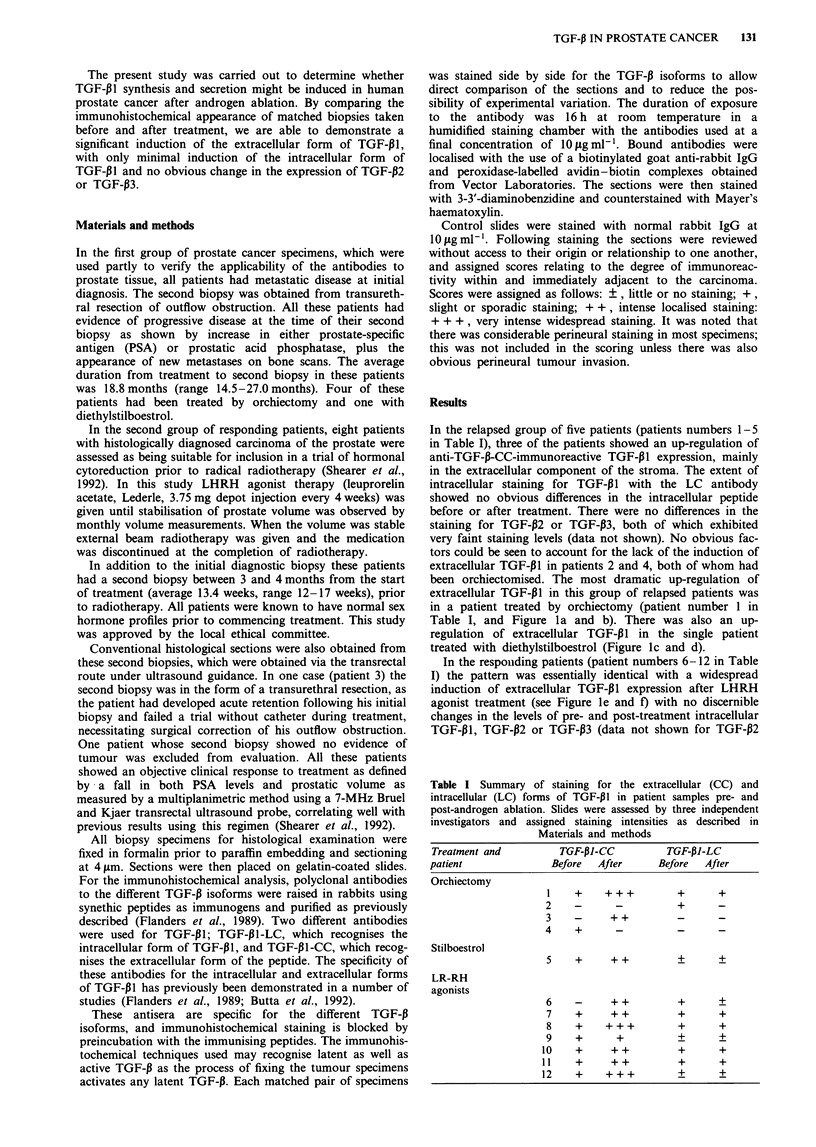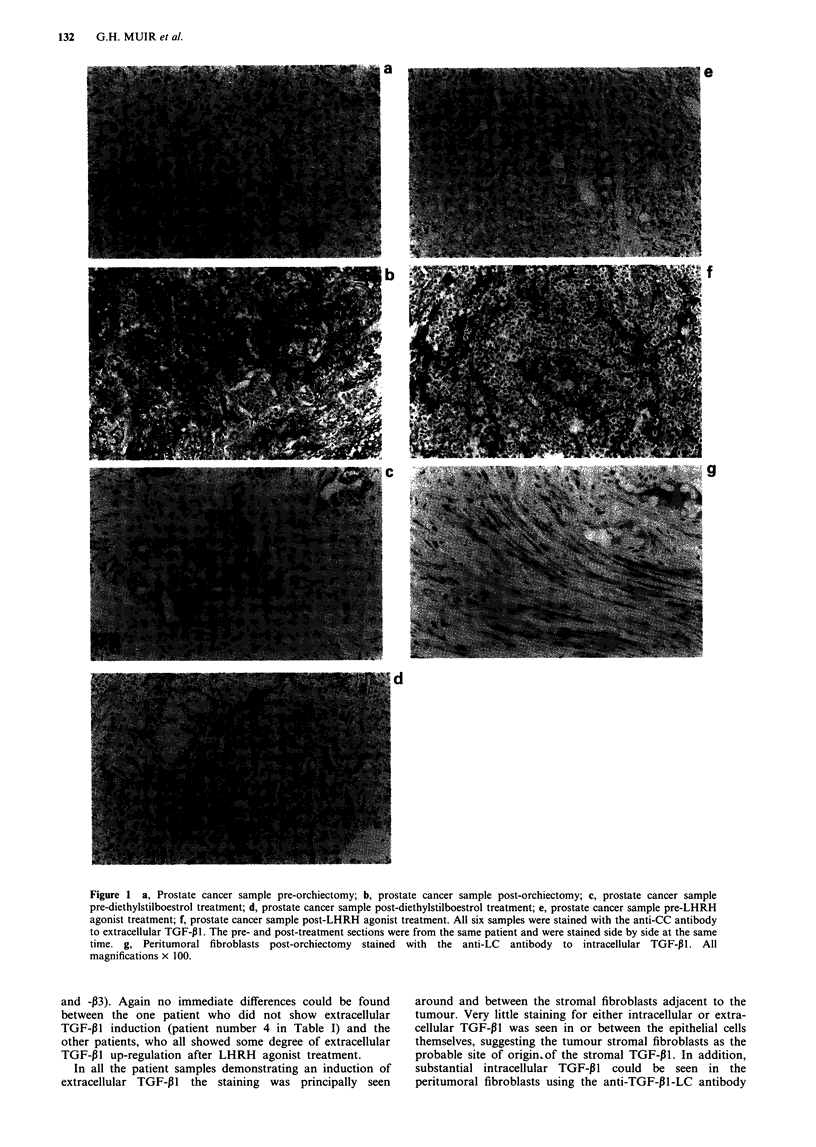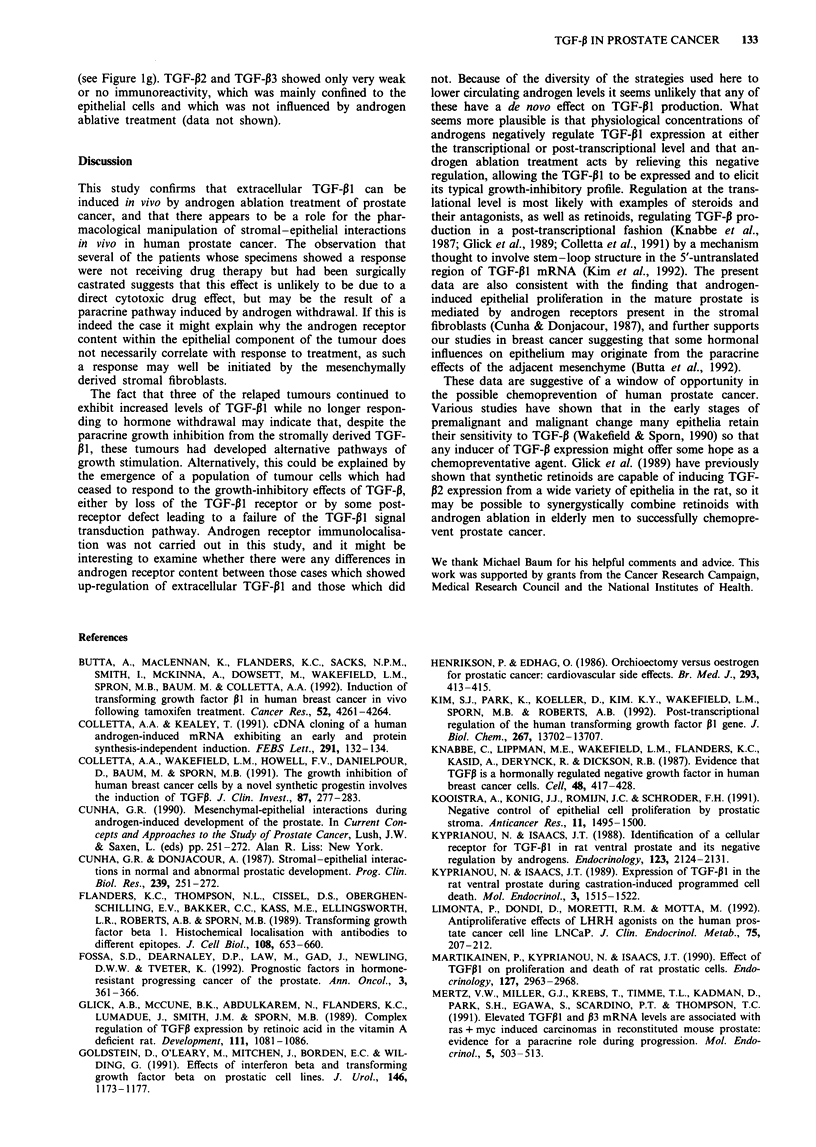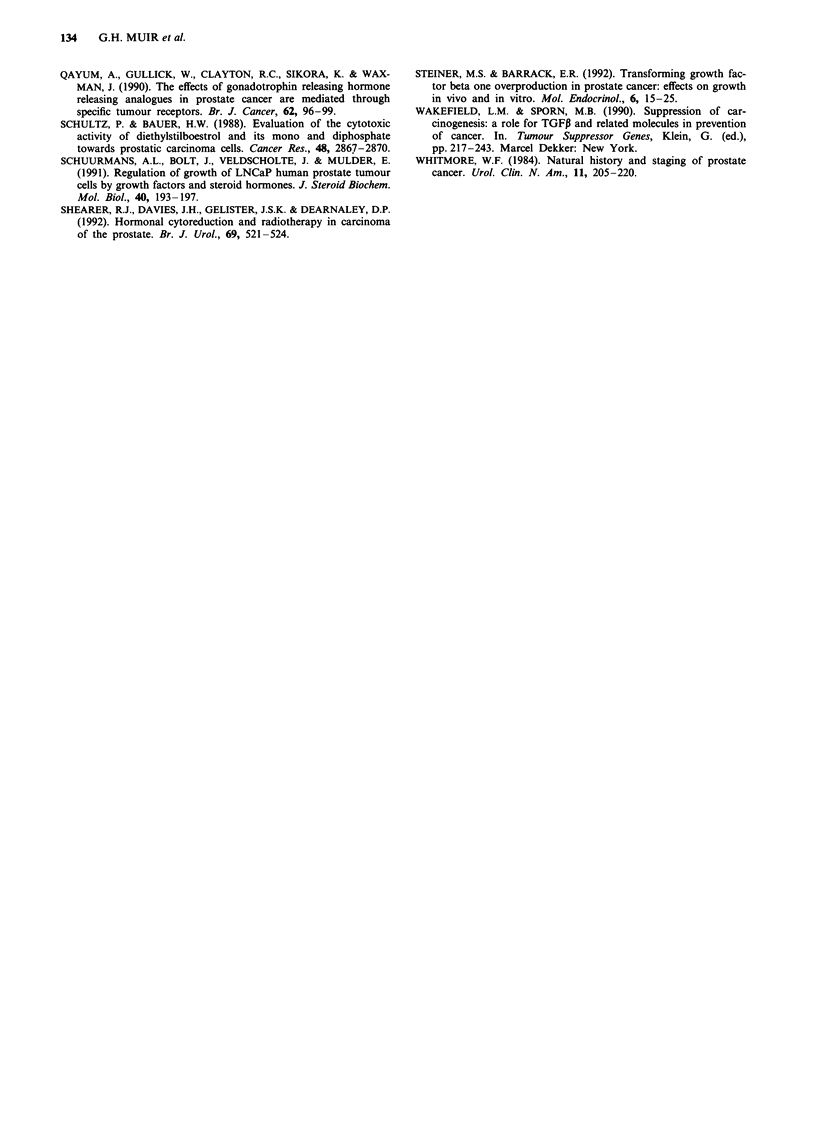# Induction of transforming growth factor beta in hormonally treated human prostate cancer.

**DOI:** 10.1038/bjc.1994.21

**Published:** 1994-01

**Authors:** G. H. Muir, A. Butta, R. J. Shearer, C. Fisher, D. P. Dearnaley, K. C. Flanders, M. B. Sporn, A. A. Colletta

**Affiliations:** Urology Unit, Institute of Cancer Research, London, UK.

## Abstract

**Images:**


Br. J. Cancer (1994), 69, 130-134 © Macmillan Press Ltd., 1994

### Induction of transforming growth factor beta in hormonally treated human prostate cancer

G.H. Muir[1], A. Butta[2], R.J. Shearer[1], C. Fisher[1], D.P. Dearnaley[1], K.C. Flanders[3], M.B. Sporn[3] & A.A. Colletta[2]

[1]*Urology Unit and* [2]*Hartwell Laboratory, Section of Academic Surgery, The Institute of Cancer Research and Royal Marsden Hospital, Fulham Road, London SW3 6JJ, UK;* [3]*Laboratory of Chemoprevention, National Cancer Institute, Bethesda, Maryland 20892, USA.*

**Summary** Transforming growth factor beta-1 (TGF-β1) has been proposed as a mediator of tumour growth in a number of tumours and cell lines including prostate, and in a recent study was shown to be up-regulated in the stroma of breast cancer tissue following treatment with the anti-oestrogen tamoxifen. Immunolocalisation of the intracellular form of TGF-β1 confirmed that the source of the stromal TGF-β1 was the peritumoral fibroblasts. We present here the results of a study in which five patients with hormonally unresponsive prostatic carcinoma and seven patients responding to a luteinising hormone-releasing hormone analogue had prostate biopsies taken before and during treatment. These were stained for TGF-β expression prior to treatment and at either relapse or 3 months later respectively. Six of seven clinically responding tumours and three of five relapsed tumours showed up-regulation of extracellular TGF-β1, again primarily in the stroma, with no apparent up-regulation of intracellular TGF-β1, TGF-β2 or TGF-β3. These data illustrate that the epithelial growth inhibitor TGF-β1 can be induced by hormonal manipulation in prostate cancer *in vivo*, and may continue to be up-regulated even after relapse. This suggests that relapse of hormonally treated prostate cancer may be associated with a failure of the epithelium to respond to stromal TGF-β1.

Carcinoma of the prostate is now the most commonly diagnosed male cancer in the USA, with an annual incidence of 86,000 cases in 1990. In the UK the reported incidence is lower, with 19.5 cases per 100,000 males reported in 1986; in both countries it is now the second most common cause of male cancer death and the incidence appears to be increasing.

The disease tends to present at an advanced stage unsuitable for radical treatment (Whitmore, 1984). Because some 80-90% of tumours are initially sensitive to androgen deprivation, hormonal manipulation, either alone or as an adjuvant therapy, is used in a large number of patients with an average response time of around 15 months. However, following relapse there is no universally effective form of treatment and the prognosis is extremely poor (Fossa *et al.*, 1992). There is no reliable method at present of predicting which tumours will not respond to treatment, and conventional assessment of the androgen receptor content of tumour cells does not seem to be of significant help. The mechanism of regression of prostate cancer in response to androgen deprivation is probably via a pathway of programmed cell death, as this has been demonstrated both in the rat ventral prostate following castration (Kyprianou & Isaacs, 1988) and in androgen-dependent LNCaP prostate cancer cells, which undergo programmed cell death in the absence of exogenous androgens (Colletta & Kealey, 1991).

Until recently the synthetic oestrogen diethylstilboestrol was a common method of medical treatment of prostate cancer, although it has fallen from favour because of its unacceptable levels of cardiovascular side-effects (Henrikson & Edhag, 1986). The majority of British urologists now use either orchiectomy or luteinising hormone-releasing hormone analogues (LHRH) in this context. Both stilboestrol (Schultz & Bauer, 1988) and LHRH analogues (Qayum *et al.*, 1990; Limonta *et al.*, 1992) are known to have directly cytotoxic effects on prostatic cancer cells *in vitro*, but the conventional view of their mode of action centres around suppression of androgen production via the hypothalamic-pituitary- gonadal axis.

The role of stromal-epithelial interactions in the developing prostate has been elegantly demonstrated by Cunha and co-workers (for example Cunha, 1990). These studies illustrated that androgen receptors in the developing prostate could be detected in the mesenchyme prior to their expression in the epithelial component of the gland. Further experiments suggested that the hormonal responsiveness of the epithelial cells in tissue recombinants was conferred by soluble factors derived from, and specific to, different types of mesenchyme. The development of male sex organs appears therefore to depend intimately on interaction between the glandular and stromal elements in the developing embryo. Proliferation of glandular tissue in the mature prostate appears to be regulated by the stromal component, implying that paracrine regulation of prostatic epithelial cells by the stroma persists throughout life. In addition, prostatic stromal cells are capable of inhibiting epithelial prostatic cancer cells in culture, which would appear to be an organ-specific finding in view of the fact that skin fibroblasts in such a system cause a stimulation of cell growth (Kooistra *et al.*, 1991).

TGF-β1 is a member of a family of tissue polypeptides that play a number of roles in the development, differentiation and growth of epithelial tissues. In most breast cancer cell types *in vitro* the TGF-βs are potent inhibitors of cellular proliferation (Knabbe *et al.*, 1987), and this has been found to be the case in human PC-3 (Goldstein *et al.*, 1991) and LNCaP cells (Schuurmans *et al.*, 1991) as well as Dunning rat prostate cancer cells *in vitro* (Steiner & Barrack, 1992). In oncogene-induced mouse prostate carcinoma and mesenchymal dysplasia, elevated levels of TGF-β1 are found, which led Mertz *et al.* (1991) to postulate that this might be due to a mesenchymal stimulation of the epithelium towards malignant change. Steiner & Barrack (1992) have also reported that clones of Dunning rat prostate cancer cells that overexpress TGF-β1 form more anaplastic and larger tumours; thus there would initially appear to be some possible conflict as to whether TGF-β1 does possess an inhibitory effect *in vivo*. Further experiments by Kyprianou and colleagues (for example Kyprianou & Isaacs, 1989) have gone a considerable way towards showing that a negative inhibitory paracrine pathway exists, by demonstrating the presence of TGF-β receptors in the rat ventral prostate, which is negatively regulated by androgens and which corresponds to a rise in TGF-β production during castration-induced programmed cell death. They have also shown that TGF-β1 can inhibit rat prostate cells *in vivo*, even with physiological levels of androgens present (Martikainen *et al.*, 1990).

Correspondence: G.H. Muir.
Received 12 May 1993; and in revised form 19 August 1993.

The present study was carried out to determine whether TGF-β1 synthesis and secretion might be induced in human prostate cancer after androgen ablation. By comparing the immunohistochemical appearance of matched biopsies taken before and after treatment, we are able to demonstrate a significant induction of the extracellular form of TGF-β1, with only minimal induction of the intracellular form of TGF-β1 and no obvious change in the expression of TGF-β2 or TGF-β3.

### Materials and methods

In the first group of prostate cancer specimens, which were used partly to verify the applicability of the antibodies to prostate tissue, all patients had metastatic disease at initial diagnosis. The second biopsy was obtained from transurethral resection of outflow obstruction. All these patients had evidence of progressive disease at the time of their second biopsy as shown by increase in either prostate-specific antigen (PSA) or prostatic acid phosphatase, plus the appearance of new metastases on bone scans. The average duration from treatment to second biopsy in these patients was 18.8 months (range 14.5–27.0 months). Four of these patients had been treated by orchiectomy and one with diethylstilboestrol.

In the second group of responding patients, eight patients with histologically diagnosed carcinoma of the prostate were assessed as being suitable for inclusion in a trial of hormonal cytoreduction prior to radical radiotherapy (Shearer *et al.*, 1992). In this study LHRH agonist therapy (leuprorelin acetate, Lederle, 3.75 mg depot injection every 4 weeks) was given until stabilisation of prostate volume was observed by monthly volume measurements. When the volume was stable external beam radiotherapy was given and the medication was discontinued at the completion of radiotherapy.

In addition to the initial diagnostic biopsy these patients had a second biopsy between 3 and 4 months from the start of treatment (average 13.4 weeks, range 12–17 weeks), prior to radiotherapy. All patients were known to have normal sex hormone profiles prior to commencing treatment. This study was approved by the local ethical committee.

Conventional histological sections were also obtained from these second biopsies, which were obtained via the transrectal route under ultrasound guidance. In one case (patient 3) the second biopsy was in the form of a transurethral resection, as the patient had developed acute retention following his initial biopsy and failed a trial without catheter during treatment, necessitating surgical correction of his outflow obstruction. One patient whose second biopsy showed no evidence of tumour was excluded from evaluation. All these patients showed an objective clinical response to treatment as defined by a fall in both PSA levels and prostatic volume as measured by a multiplanimetric method using a 7-MHz Bruel and Kjaer transrectal ultrasound probe, correlating well with previous results using this regimen (Shearer *et al.*, 1992).

All biopsy specimens for histological examination were fixed in formalin prior to paraffin embedding and sectioning at 4 μm. Sections were then placed on gelatin-coated slides. For the immunohistochemical analysis, polyclonal antibodies to the different TGF-β isoforms were raised in rabbits using synethic peptides as immunogens and purified as previously described (Flanders *et al.*, 1989). Two different antibodies were used for TGF-β1; TGF-β1-LC, which recognises the intracellular form of TGF-β1, and TGF-β1-CC, which recognises the extracellular form of the peptide. The specificity of these antibodies for the intracellular and extracellular forms of TGF-β1 has previously been demonstrated in a number of studies (Flanders *et al.*, 1989; Butta *et al.*, 1992).

These antisera are specific for the different TGF-β isoforms, and immunohistochemical staining is blocked by preincubation with the immunising peptides. The immunohistochemical techniques used may recognise latent as well as active TGF-β as the process of fixing the tumour specimens activates any latent TGF-β. Each matched pair of specimens was stained side by side for the TGF-β isoforms to allow direct comparison of the sections and to reduce the possibility of experimental variation. The duration of exposure to the antibody was 16 h at room temperature in a humidified staining chamber with the antibodies used at a final concentration of 10 μg ml$^{-1}$. Bound antibodies were localised with the use of a biotinylated goat anti-rabbit IgG and peroxidase-labelled avidin–biotin complexes obtained from Vector Laboratories. The sections were then stained with 3-3′-diaminobenzidine and counterstained with Mayer's haematoxylin.

Control slides were stained with normal rabbit IgG at 10 μg ml$^{-1}$. Following staining the sections were reviewed without access to their origin or relationship to one another, and assigned scores relating to the degree of immunoreactivity within and immediately adjacent to the carcinoma. Scores were assigned as follows: ±, little or no staining; +, slight or sporadic staining; + +, intense localised staining: + + +, very intense widespread staining. It was noted that there was considerable perineural staining in most specimens; this was not included in the scoring unless there was also obvious perineural tumour invasion.

### Results

In the relapsed group of five patients (patients numbers 1–5 in Table I), three of the patients showed an up-regulation of anti-TGF-β-CC-immunoreactive TGF-β1 expression, mainly in the extracellular component of the stroma. The extent of intracellular staining for TGF-β1 with the LC antibody showed no obvious differences in the intracellular peptide before or after treatment. There were no differences in the staining for TGF-β2 or TGF-β3, both of which exhibited very faint staining levels (data not shown). No obvious factors could be seen to account for the lack of the induction of extracellular TGF-β1 in patients 2 and 4, both of whom had been orchiectomised. The most dramatic up-regulation of extracellular TGF-β1 in this group of relapsed patients was in a patient treated by orchiectomy (patient number 1 in Table I, and Figure 1a and b). There was also an up-regulation of extracellular TGF-β1 in the single patient treated with diethylstilboestrol (Figure 1c and d).

In the responding patients (patient numbers 6–12 in Table I) the pattern was essentially identical with a widespread induction of extracellular TGF-β1 expression after LHRH agonist treatment (see Figure 1e and f) with no discernible changes in the levels of pre- and post-treatment intracellular TGF-β1, TGF-β2 or TGF-β3 (data not shown for TGF-β2

**Table I** Summary of staining for the extracellular (CC) and intracellular (LC) forms of TGF-β1 in patient samples pre- and post-androgen ablation. Slides were assessed by three independent investigators and assigned staining intensities as described in Materials and methods

| *Treatment and patient* | | *TGF-β1-CC* | | *TGF-β1-LC* | |
|---|---|---|---|---|---|
| | | *Before* | *After* | *Before* | *After* |
| Orchiectomy | | | | | |
| | 1 | + | + + + | + | + |
| | 2 | – | – | + | – |
| | 3 | – | + + | – | – |
| | 4 | + | – | – | – |
| Stilboestrol | | | | | |
| | 5 | + | + + | ± | ± |
| LR-RH agonists | | | | | |
| | 6 | – | + + | + | ± |
| | 7 | + | + + | + | + |
| | 8 | + | + + + | + | + |
| | 9 | + | + | ± | ± |
| | 10 | + | + + | + | + |
| | 11 | + | + + | + | + |
| | 12 | + | + + + | ± | ± |

**Figure 1** **a**, Prostate cancer sample pre-orchiectomy; **b**, prostate cancer sample post-orchiectomy; **c**, prostate cancer sample pre-diethylstilboestrol treatment; **d**, prostate cancer sample post-diethylstilboestrol treatment; **e**, prostate cancer sample pre-LHRH agonist treatment; **f**, prostate cancer sample post-LHRH agonist treatment. All six samples were stained with the anti-CC antibody to extracellular TGF-β1. The pre- and post-treatment sections were from the same patient and were stained side by side at the same time. **g**, Peritumoral fibroblasts post-orchiectomy stained with the anti-LC antibody to intracellular TGF-β1. All magnifications × 100.

and -β3). Again no immediate differences could be found between the one patient who did not show extracellular TGF-β1 induction (patient number 4 in Table I) and the other patients, who all showed some degree of extracellular TGF-β1 up-regulation after LHRH agonist treatment.

In all the patient samples demonstrating an induction of extracellular TGF-β1 the staining was principally seen around and between the stromal fibroblasts adjacent to the tumour. Very little staining for either intracellular or extracellular TGF-β1 was seen in or between the epithelial cells themselves, suggesting the tumour stromal fibroblasts as the probable site of origin of the stromal TGF-β1. In addition, substantial intracellular TGF-β1 could be seen in the peritumoral fibroblasts using the anti-TGF-β1-LC antibody

(see Figure 1g). TGF-β2 and TGF-β3 showed only very weak or no immunoreactivity, which was mainly confined to the epithelial cells and which was not influenced by androgen ablative treatment (data not shown).

### Discussion

This study confirms that extracellular TGF-β1 can be induced *in vivo* by androgen ablation treatment of prostate cancer, and that there appears to be a role for the pharmacological manipulation of stromal–epithelial interactions *in vivo* in human prostate cancer. The observation that several of the patients whose specimens showed a response were not receiving drug therapy but had been surgically castrated suggests that this effect is unlikely to be due to a direct cytotoxic drug effect, but may be the result of a paracrine pathway induced by androgen withdrawal. If this is indeed the case it might explain why the androgen receptor content within the epithelial component of the tumour does not necessarily correlate with response to treatment, as such a response may well be initiated by the mesenchymally derived stromal fibroblasts.

The fact that three of the relaped tumours continued to exhibit increased levels of TGF-β1 while no longer responding to hormone withdrawal may indicate that, despite the paracrine growth inhibition from the stromally derived TGF-β1, these tumours had developed alternative pathways of growth stimulation. Alternatively, this could be explained by the emergence of a population of tumour cells which had ceased to respond to the growth-inhibitory effects of TGF-β, either by loss of the TGF-β1 receptor or by some post-receptor defect leading to a failure of the TGF-β1 signal transduction pathway. Androgen receptor immunolocalisation was not carried out in this study, and it might be interesting to examine whether there were any differences in androgen receptor content between those cases which showed up-regulation of extracellular TGF-β1 and those which did not. Because of the diversity of the strategies used here to lower circulating androgen levels it seems unlikely that any of these have a *de novo* effect on TGF-β1 production. What seems more plausible is that physiological concentrations of androgens negatively regulate TGF-β1 expression at either the transcriptional or post-transcriptional level and that androgen ablation treatment acts by relieving this negative regulation, allowing the TGF-β1 to be expressed and to elicit its typical growth-inhibitory profile. Regulation at the translational level is most likely with examples of steroids and their antagonists, as well as retinoids, regulating TGF-β production in a post-transcriptional fashion (Knabbe *et al.*, 1987; Glick *et al.*, 1989; Colletta *et al.*, 1991) by a mechanism thought to involve stem–loop structure in the 5′-untranslated region of TGF-β1 mRNA (Kim *et al.*, 1992). The present data are also consistent with the finding that androgen-induced epithelial proliferation in the mature prostate is mediated by androgen receptors present in the stromal fibroblasts (Cunha & Donjacour, 1987), and further supports our studies in breast cancer suggesting that some hormonal influences on epithelium may originate from the paracrine effects of the adjacent mesenchyme (Butta *et al.*, 1992).

These data are suggestive of a window of opportunity in the possible chemoprevention of human prostate cancer. Various studies have shown that in the early stages of premalignant and malignant change many epithelia retain their sensitivity to TGF-β (Wakefield & Sporn, 1990) so that any inducer of TGF-β expression might offer some hope as a chemopreventative agent. Glick *et al.* (1989) have previously shown that synthetic retinoids are capable of inducing TGF-β2 expression from a wide variety of epithelia in the rat, so it may be possible to synergystically combine retinoids with androgen ablation in elderly men to successfully chemoprevent prostate cancer.

We thank Michael Baum for his helpful comments and advice. This work was supported by grants from the Cancer Research Campaign, Medical Research Council and the National Institutes of Health.

### References

BUTTA, A., MACLENNAN, K., FLANDERS, K.C., SACKS, N.P.M., SMITH, I., MCKINNA, A., DOWSETT, M., WAKEFIELD, L.M., SPRON, M.B., BAUM. M. & COLLETTA, A.A. (1992). Induction of transforming growth factor β1 in human breast cancer in vivo following tamoxifen treatment. *Cancer Res.*, **52**, 4261–4264.

COLLETTA, A.A. & KEALEY, T. (1991). cDNA cloning of a human androgen-induced mRNA exhibiting an early and protein synthesis-independent induction. *FEBS Lett.*, **291**, 132–134.

COLLETTA, A.A., WAKEFIELD, L.M., HOWELL, F.V., DANIELPOUR, D., BAUM, M. & SPORN, M.B. (1991). The growth inhibition of human breast cancer cells by a novel synthetic progestin involves the induction of TGFβ. *J. Clin. Invest.*, **87**, 277–283.

CUNHA, G.R. (1990). Mesenchymal-epithelial interactions during androgen-induced development of the prostate. In *Current Concepts and Approaches to the Study of Prostate Cancer*, Lush, J.W. & Saxen, L. (eds) pp. 251–272. Alan R. Liss: New York.

CUNHA, G.R. & DONJACOUR, A. (1987). Stromal–epithelial interactions in normal and abnormal prostatic development. *Prog. Clin. Biol. Res.*, **239**, 251–272.

FLANDERS, K.C., THOMPSON, N.L., CISSEL, D.S., OBERGHEN-SCHILLING, E.V., BAKKER, C.C., KASS, M.E., ELLINGSWORTH, L.R., ROBERTS, A.B. & SPORN, M.B. (1989). Transforming growth factor beta 1. Histochemical localisation with antibodies to different epitopes. *J. Cell Biol.*, **108**, 653–660.

FOSSA, S.D., DEARNALEY, D.P., LAW, M., GAD, J., NEWLING, D.W.W. & TVETER, K. (1992). Prognostic factors in hormone-resistant progressing cancer of the prostate. *Ann. Oncol.*, **3**, 361–366.

GLICK, A.B., MCCUNE, B.K., ABDULKAREM, N., FLANDERS, K.C., LUMADUE, J., SMITH, J.M. & SPORN, M.B. (1989). Complex regulation of TGFβ expression by retinoic acid in the vitamin A deficient rat. *Development*, **111**, 1081–1086.

GOLDSTEIN, D., O'LEARY, M., MITCHEN, J., BORDEN, E.C. & WILDING, G. (1991). Effects of interferon beta and transforming growth factor beta on prostatic cell lines. *J. Urol.*, **146**, 1173–1177.

HENRIKSON, P. & EDHAG, O. (1986). Orchioectomy versus oestrogen for prostatic cancer: cardiovascular side effects. *Br. Med. J.*, **293**, 413–415.

KIM, S.J., PARK, K., KOELLER, D., KIM. K.Y., WAKEFIELD, L.M., SPORN, M.B. & ROBERTS, A.B. (1992). Post-transcriptional regulation of the human transforming growth factor β1 gene. *J. Biol. Chem.*, **267**, 13702–13707.

KNABBE, C., LIPPMAN, M.E., WAKEFIELD, L.M., FLANDERS, K.C., KASID, A., DERYNCK, R. & DICKSON, R.B. (1987). Evidence that TGFβ is a hormonally regulated negative growth factor in human breast cancer cells. *Cell*, **48**, 417–428.

KOOISTRA, A., KONIG, J.J., ROMIJN, J.C. & SCHRODER, F.H. (1991). Negative control of epithelial cell proliferation by prostatic stroma. *Anticancer Res.*, **11**, 1495–1500.

KYPRIANOU, N. & ISAACS, J.T. (1988). Identification of a cellular receptor for TGF-β1 in rat ventral prostate and its negative regulation by androgens. *Endocrinology*, **123**, 2124–2131.

KYPRIANOU, N. & ISAACS, J.T. (1989). Expression of TGF-β1 in the rat ventral prostate during castration-induced programmed cell death. *Mol. Endocrinol.*, **3**, 1515–1522.

LIMONTA, P., DONDI, D., MORETTI, R.M. & MOTTA, M. (1992). Antiproliferative effects of LHRH agonists on the human prostate cancer cell line LNCaP. *J. Clin. Endocrinol. Metab.*, **75**, 207–212.

MARTIKAINEN, P., KYPRIANOU, N. & ISAACS, J.T. (1990). Effect of TGFβ1 on proliferation and death of rat prostatic cells. *Endocrinology*, **127**, 2963–2968.

MERTZ, V.W., MILLER, G.J., KREBS, T., TIMME, T.L., KADMAN, D., PARK, S.H., EGAWA, S., SCARDINO, P.T. & THOMPSON, T.C. (1991). Elevated TGFβ1 and β3 mRNA levels are associated with ras + myc induced carcinomas in reconstituted mouse prostate: evidence for a paracrine role during progression. *Mol. Endocrinol.*, **5**, 503–513.

QAYUM, A., GULLICK, W., CLAYTON, R.C., SIKORA, K. & WAXMAN, J. (1990). The effects of gonadotrophin releasing hormone releasing analogues in prostate cancer are mediated through specific tumour receptors. *Br. J. Cancer*, **62**, 96–99.

SCHULTZ, P. & BAUER, H.W. (1988). Evaluation of the cytotoxic activity of diethylstilboestrol and its mono and diphosphate towards prostatic carcinoma cells. *Cancer Res.*, **48**, 2867–2870.

SCHUURMANS, A.L., BOLT, J., VELDSCHOLTE, J. & MULDER, E. (1991). Regulation of growth of LNCaP human prostate tumour cells by growth factors and steroid hormones. *J. Steroid Biochem. Mol. Biol.*, **40**, 193–197.

SHEARER, R.J., DAVIES, J.H., GELISTER, J.S.K. & DEARNALEY, D.P. (1992). Hormonal cytoreduction and radiotherapy in carcinoma of the prostate. *Br. J. Urol.*, **69**, 521–524.

STEINER, M.S. & BARRACK, E.R. (1992). Transforming growth factor beta one overproduction in prostate cancer: effects on growth in vivo and in vitro. *Mol. Endocrinol.*, **6**, 15–25.

WAKEFIELD, L.M. & SPORN, M.B. (1990). Suppression of carcinogenesis: a role for TGFβ and related molecules in prevention of cancer. In. *Tumour Suppressor Genes*, Klein, G. (ed.), pp. 217–243. Marcel Dekker: New York.

WHITMORE, W.F. (1984). Natural history and staging of prostate cancer. *Urol. Clin. N. Am.*, **11**, 205–220.